# Comparison of immunogenicity and safety outcomes of a malaria vaccine FMP013/ALFQ in rhesus macaques (*Macaca mulatta*) of Indian and Chinese origin

**DOI:** 10.1186/s12936-019-3014-5

**Published:** 2019-11-27

**Authors:** Monica L. Martin, Alexis A. Bitzer, Andrew Schrader, Elke S. Bergmann-Leitner, Kim Soto, Xiaoyan Zou, Zoltan Beck, Gary R. Matyas, Sheetij Dutta

**Affiliations:** 1 Division of Veterinary Medicine, Walter Reed Army Institute of Research, Silver Spring, MD 20910 USA; 2 Structural Biologics Laboratory, Malaria Biologics Branch, Walter Reed Army Institute of Research, Silver Spring, MD 20910 USA; 3 Immunology Core, Malaria Biologics Branch, Walter Reed Army Institute of Research, Silver Spring, MD 20910 USA; 4 Malaria Department, Naval Medical Research Center, Silver Spring, MD 20910 USA; 5 Military HIV Research Program, Walter Reed Army Institute of Research, Silver Spring, MD 20910 USA; 6 Henry M. Jackson Foundation, Rockville, MD 20852 USA

**Keywords:** Circumsporozoite protein, CSP, Rhesus, Chinese origin, Indian origin, Macaque, Malaria, ALFQ, FMP013

## Abstract

**Background:**

Indian-origin rhesus (InR) are preferred for research, but strict export restrictions continue to limit their use. Chinese-origin rhesus (ChR), although easier to procure, are genetically distinct from InR and differ in their immune response to infectious agents, such as the Simian Immunodeficiency Virus. The most advanced malaria vaccine, RTS,S (GlaxoSmithKline), is based on the circumsporozoite protein (CSP) of *Plasmodium falciparum*. The efficacy of RTS,S vaccine in the field remains low and short-lived; efforts are underway to improve CSP-based vaccines. Rhesus models can accelerate preclinical down-selection of the next generation of malaria vaccines. This study was used to determine if the safety and immunogenicity outcomes following vaccination with a CSP vaccine would differ in the InR and ChR models, given the genetic differences between the two sub-populations of rhesus.

**Methods:**

The FMP013 vaccine, was composed of nearly full-length soluble *P. falciparum* CSP produced in *Escherichia coli* and was adjuvanted with the Army liposomal formulation (ALFQ). Three doses of the vaccine were administered in InR and ChR (n = 6) at 1-month intervals and the antibody and T cell responses were assessed.

**Results:**

Local and systemic toxicity profile of FMP013 vaccine in InR and ChR were similar and they revealed that the FMP013 vaccine was safe and caused only mild and transient inflammatory adverse reactions. Following the first 2 vaccines, there was a slower acquisition of antibodies to the CSP repeat region in ChR. However after the 3rd vaccination the titers in the two models were comparable. The ChR group repeat-specific antibodies had higher avidity and ChR group showed higher inhibition of liver stage development activity compared to InR. There was no difference in T-cell responses to the FMP013 vaccine between the two models.

**Conclusions:**

A difference in the quality of serological responses was detected between the two sub-populations of rhesus. However, both models confirmed that FMP013/ALFQ vaccine was safe, highly immunogenic, elicited functional antibodies and T-cell responses. Overall, the data suggests that rhesus of Indian and Chinese origins can be interchangeably used to compare the safety and immunogenicity of next-generation of malaria vaccines and adjuvants.

## Background

Malaria infects over 200 million individuals every year, and despite the reduction in morbidity and mortality in the last 20 years, more than 400,000 deaths are reported annually [1, 2].

*Plasmodium falciparum* is one of the species that is most commonly associated with the severe and fatal form of malaria that is prevalent in sub-Saharan Africa [1]. RTS,S (GlaxoSmithKline Vaccines, Rixensart, Belgium) is a recombinant malaria antigen based on the circumsporozoite protein (CSP) of *P. falciparum*. The CSP portion of RTS,S consists of 19 NPNA repeating units and the C-terminal region while the remainder of the protein is the hepatitis B surface antigen [3, 4]. While, the NPNA repeats are highly conserved across all *P. falciparum* strains, the C-terminal region contains polymorphic residues, which could be one of the reasons why a monovalent CSP vaccine, such as RTS,S, confers partial protection against diverse parasite strains prevalent in endemic areas [5]. RTS,S is formulated in the potent adjuvant AS01B, that contains two immune-stimulators: monophosphoryl-lipid A and QS-21. Vaccination with RTS,S/AS01 induces high level of protection against controlled human malaria infection however, in Phase 3 trials, RTS,S/AS01 induces less than 50% protection against natural infection [6, 7]. In 2015, a paediatric formulation of RTS,S/AS01E (Mosquirix™) received regulatory approval, and is in pilot studies in three African countries, to determine its effectiveness in malaria control when deployed by the public health system [8].

As a step towards improving the efficacy of RTS,S/AS01, Walter Reed Army Institute of Research has developed a CSP-based antigen, FMP013, which is a soluble protein vaccine aimed at broadening the immunity to epitopes not present within the RTS,S construct as it also contains the N-terminal region along with junctional epitopes and minor repeats of CSP [9, 10]. FMP013 was formulated in a potent adjuvant, the Army Liposomal Formulation containing QS-21 (ALFQ). The immune-stimulants present within ALFQ, 3D-PHAD™ (TLR-4 agonist) and QS-21 (modulator of innate immunity) have been shown to be critical for the optimal immunogenicity of FMP013 vaccine in mice and rhesus models [10–12].

Since first reported in 1995, it took 15 years and millions of dollars to conduct the series of clinical trials that led to recently initiated pilot implementation of Mosquirix™, in Africa [13]. With limited resources and high regulatory costs, a next-generation malaria vaccine would need to rely heavily upon animal models to accelerate progression. Mouse models are excellent for understanding how innate and acquired immunity against the malaria parasite protects and there are transgenic parasites that allow the evaluation of protective efficacy of human malaria vaccines in mice [14]. Mouse models, although useful, are not a reliable predictor of human malaria vaccine outcomes as was shown in a mouse and rhesus comparative immunogenicity study using two CSP-based vaccines [15]. Rhesus model also has limitations, as rhesus-specific immunological reagents are not well characterized; yet it is argued that for vaccine candidates, such as CSP, rhesus ought to remain on the critical path of de-risking second-generation CSP formulations before transitioning to human vaccines trials [15]. Historically, the Indian origin rhesus (InR) has been a preferred model for research based on the published data [16, 17]. However, due to a 1978 ban on exporting rhesus [18], InR research in the United States, has relied exclusively on purpose-bred colonies.

An alternative to InR are wild-caught and colony-bred rhesus of Chinese origin (ChR). ChR males are heavier, longer, and taller than InR males [19, 20], and they differ from InR in behaviour, physiology and temperament [19, 21]. At the genetic level, these two populations have been differentiated by single nucleotide polymorphisms and mitochondrial DNA sequences [18, 22]. ChR populations are known to be more genetically diverse than InR [23] and this difference has been shown to affect some disease and vaccine outcomes between the two models. For example, simian immunodeficiency virus (SIV) infected ChR had higher CD4+ cell counts and lower viral loads than InR [23, 24]. ChR are believed to better mimic the slower progression of human immunodeficiency virus (HIV) infection in humans [25] and have become useful models for HIV vaccine research [23, 26]. Among other factors, the difference in SIV infection profile has been correlated with a difference in the copy number of an HIV-suppressive chemokine CCL3L and a more Th1-biased T cell response in ChR [27, 28].

In light of easier access and availability, malaria vaccine developers would need to increasingly rely on the ChR model. To determine if these observed differences in SIV infection profile also extended to malaria vaccine outcomes, the FMP013 vaccine adjuvanted with ALFQ was compared head-to-head in the InR and ChR models. Important differences were observed in antibody quality between the two models. While these immunological differences between the two models need to be factored into the design and interpretation of future rhesus trials, the data suggests that ChR and InR could be used interchangeably for malaria vaccine down-selection studies.

## Methods

### Rhesus

Adult rhesus macaques of Indian (InR) and Chinese (ChR) origin, were housed at the Walter Reed Army Institute of Research (WRAIR) animal facility, and used under an IACUC-approved protocol. InR were colony bred in Alice, Texas, their ages ranged from 8 to 11 years old. The ChR, also in the 8–12 years age-range, were imported from China over several months from March 2013 to November 2013. Capture sites or breeding sites of ChR were unknown. ChR were housed in separate rooms from the InR and all monkeys were tested sero-negative for macacine herpesvirus 1, measles, simian retrovirus, simian immunodeficiency virus (SIV), simian T cell leukemia virus, and tuberculin skin test. Additionally, all macaques were pre-screened for pre-existing antibodies against *P. falciparum* CSP and antibodies against the immune-stimulator monophosphoryl lipid-A. Animals were pair-housed with same sex and conspecific origins, fed a commercial diet (Lab Diet 5038, Purina Mills International), provided water ad libitum, and supplemented with a variety of fresh fruits and vegetables. Environmental enrichment was provided in accordance with WRAIR Veterinary Service Program standard operating procedures. Animal cages were cleaned daily and sanitized bimonthly. Automatic lighting was on a 12:12 h cycle.

### Vaccines and adjuvant

The FMP013 was cGMP grade soluble *Escherichia coli* product containing the *P. falciparum* CSP N-terminal region, 19 NPNA and 3 NVDP repeats and the C-terminal region [11]. Vaccine was adjuvanted with 1 mL of ALFQ containing: 200 µg 3D-PHAD™ (Avanti Polar Lipids, Alabaster, CA) and 100 µg QS-21 (Desert King, San Diego, CA) immune modulators [11]. The formulation was mixed on a rotary platform for 1 h prior to administration.

### Vaccination

Age, sex, and weight of the monkeys were approximately matched across groups (Additional file 1: Table S1). Six ChR were all females and InR had 3 males and 3 females. Animals were sedated using Ketamine 11 mg/kg and Acepromazine 0.55 mg/kg. Once sedated the vaccine injection site was shaved, cleaned and disinfected with 70% isopropyl alcohol. The InR and ChR (n = 6) received three vaccines of 40 µg FMP013, intramuscular in alternating left and right thigh, on days 0, 28, and 60.

### Safety and tolerability

Animals were sedated for all exams and blood collections. Heart rate, respiration, body weights, and rectal temperatures were recorded on days 1, 3, 7, 14, 28 (D1, D3, D7, D14, and D28) post vaccine. Injection sites were examined and compared to baseline at D1, D3, and D7 post vaccine administration. For immunization site reactogenicity, a grading scale (1 = diffuse pink or mild swelling or mild, 2 = diffuse red flush or moderate swelling or moderate, 3 = marked red flush) was used [29]. Blood samples were taken 2 weeks before the first dose and then on D1, D3, D7 for toxicology (Additional file 1: Table S2A, B). Sera were collected for ELISA at 2 week following each vaccination. All samples were collected from the femoral vein using a Vacutainer™ tubes (Becton–Dickinson, Franklin Lakes, NJ).

### ELISA

Direct and avidity ELISA were performed by the International Malaria Serology Reference Center (WRAIR, Silver Spring, MD) against the FMP013 and (NANP)_6_C repeat peptide [14]. Secondary antibody used was HRP conjugated goat anti-human. ELISA titer was defined as the serum dilution that resulted in optical density (OD) of 1.0 as predicted by a four-parameter curve fitting equation (Biotek, Winooski, VT). Avidity ELISA was conducted similar to above using 4 M urea wash for 10 min following the incubation of the primary antibody to remove low affinity antibodies. ELISA plates were developed and avidity index was calculated as the ratio of titers obtained after washing the plate with urea or with PBS [30].

### ILSDA

Whole serum was tested by inhibition of sporozoite development assay, ILSDA [14]. Briefly, the *P. falciparum* NF54 strain sporozoites obtained from salivary gland dissections of infected *Anopheles* mosquitoes were mixed with a positive control monoclonal antibody NFS1 or polyclonal rhesus serum at two dilutions (1:200 and 1:300) and incubated at room temperature for 20 min. The sporozoite-antibody mixtures were then introduced into the wells containing cryopreserved human hepatocytes (BioReclamation IVT, Baltimore, MD) and incubated at 37 °C for 3 h to allow sporozoites to infect hepatocytes. After the 3-h incubation period, hepatocytes were washed with fresh culture media to remove non-invaded sporozoites and cells were incubated at 37 °C for 96 h. The RNA from the cells was harvested and quantitative real-time PCR (qRT-PCR) analysis on Pf 18s rRNA levels was used to determine the level of inhibition of liver stage development.

### Fluorospot assay

Antigen-specific interferon (IFN)-γ, interleukin 2 (IL-2) and Tumour Necrosis Factor (TNF) cytokine-secreting T cells were quantified by Fluorospot (Ucytech Biosciences, Utrecht, Netherlands) following the manufacturer’s instructions. Monkey anti-CD3 mAb (Mabtech Inc., Cincinnati, OH) was used as an internal positive control. Each well contained 25 µL CD28 and CD49d (BD Biosciences, San Diego, CA) cell stimulants, 25 µL of antigen and 50 µL of cells (2.5 × 10^5^/well). Antigen-specific responses were stimulated with either recombinant FMP013 (10 µg/mL) or a CSP-peptide pool (16-mer peptides overlapping by 11 AA; CSP AA 97-283; 1.25 µg/mL). Plates were incubated at 37 °C, 5% CO_2_, for 40 to 48 h. Fluorospot plates were analyzed using the Autoimmun Diagnostica (AID) GmbH Fluorospot reader (Strassberg, Germany) equipped with filters for FITC (excitation 490 nm/emission 510 nm) and Cy3 (excitation 550 nm/emission 570 nm).

### Statistical analysis

Local reactions were scored on 0–3 scale and significant increase above 0 was determined by a one-tailed one sample *t*-test. Systemic reactions were compared by ANOVA followed by Dunnett’s correction for multiple comparisons. ELISA data were log transformed and multiple comparisons were made by ANOVA with p-values corrected by Tukey’s method (GraphPad Prism software, La Jolla, CA). Statistically significant difference in group means was indicated in figures as **** (p < 0.0001), *** (p < 0.001), ** (p < 0.01), or * (p < 0.05). Positive T-cell response was determined by an unpaired T-test against the respective pre-immune control.

## Results

A nearly full-length soluble protein (FMP013) was used to design a study to compare toxicological, serological and cellular immunity outcomes of CSP vaccination in rhesus macaques originating from Indian and Chinese sub-populations.

### Local reactogenicity

**Fig. 1:**
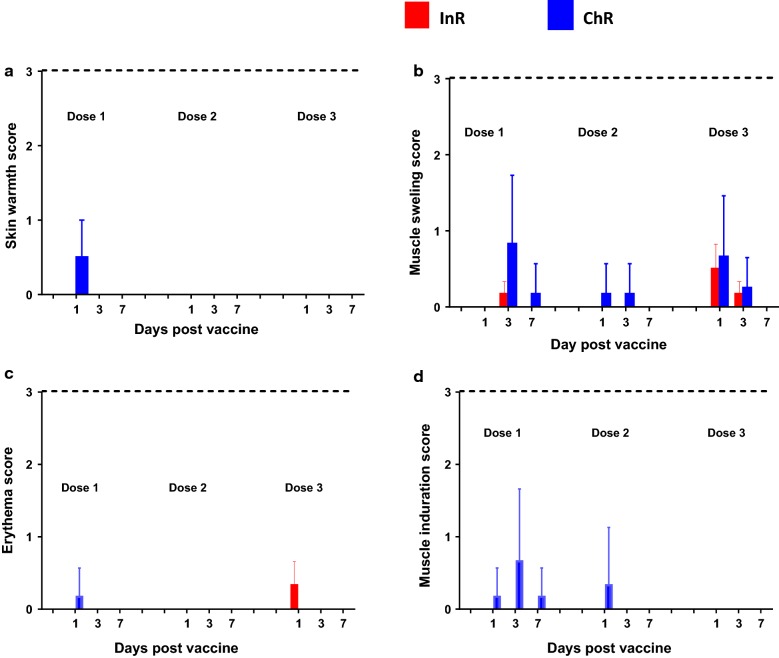
Local reactogenicity: All local adverse effects scored on 0–3 scale at days 1, 3, 7 post vaccination (x-axis). Mean  +  SEM score for local adverse events (n  =  6); dotted line represents max possible score. Bars were omitted if no reaction was observed. **a** Skin warmth, **b** muscle swelling, **c** erythema, **d** muscle induration. No group mean score was significantly above 0

There was no sub-population specific difference between the InR and ChR with respect to the systemic and local reactions to the FMP013 formulated in ALFQ. Specifically, animal body weights remained stable throughout the study. There were no signs of ulcers or abscesses and only minimal, skin warmth, erythema and muscle indurations were caused by three doses of FMP013 vaccine in InR or ChR (Fig. 1a, c, d). A mild and transient muscle swelling, was observed on D1, D3 after vaccination in both the InR and ChR, which resolved by D7 (Fig. 1b).

### Systemic toxicity

**Fig. 2:**
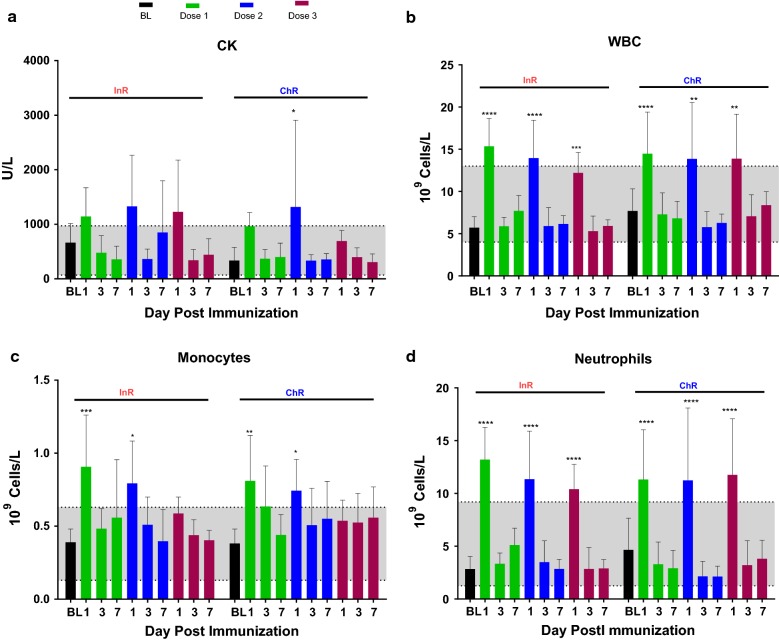
Systemic toxicity: mean  +  SEM values for blood chemistry and complete blood count on days 1, 3, 7 (n  =  6). 1st, 2nd and 3rd vaccine doses are shown in green, blue and red bars respectively. Mean baseline (BL) for each group and reference range is shaded area. **a** Creatine kinase (CK), **b** WBC, **c** monocytes and **d** neutrophils

Baseline body temperatures for all animals averaged between 100 and 102.6 °F and only one episode of fever in the ChR group (> 104 °F) was recorded out of 36 vaccinations, which resolved by D7 post vaccination. Blood biochemistry parameters as well as haematological parameters at baseline were comparable between the two sub-populations. On D1, D3 and D7 post vaccination no major changes in liver or kidney function tests were recorded in ChR and InR. A trend of increased creatine kinase (CK) on D1 post vaccination was seen in InR and ChR **(**Fig. 2a), which returned back to normal levels by D7. Blood cell count showed RBC, reticulocyte, and lymphocytes remained stable. Platelets trended to be higher by D7 post vaccination in both groups, although this rise was not significant and resolved prior to the next vaccine time-point. There was an increase in white blood cell, monocyte and neutrophil cell counts on D1 post vaccination (Fig. 2b–d). All cell count elevations were replicated by InR and ChR and these values returned back to normal by D7. Overall, InR and ChR showed no difference in local or systemic adverse events following three doses of FMP013 vaccine formulated in ALFQ.

### Antibody titer and function

**Fig. 3:**
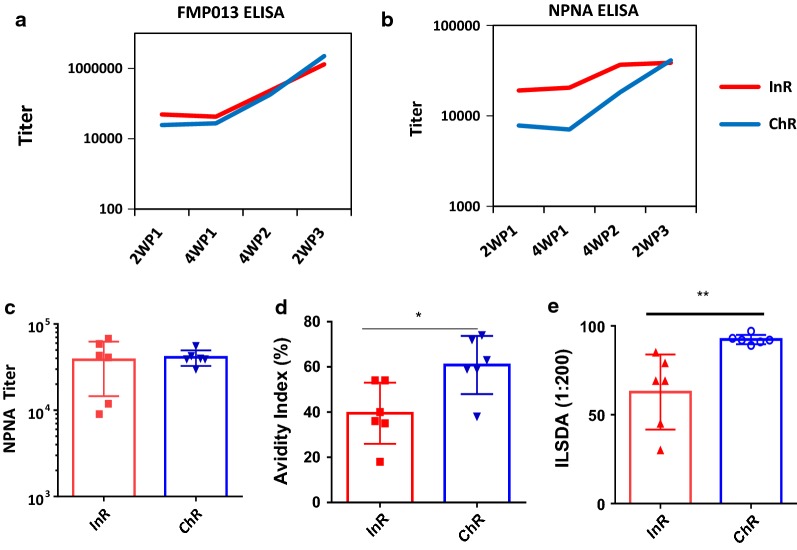
Antibody titer and function: mean ELISA titers at 2 weeks post 1st (2WP1), 4WP1, 4WP2 and 2WP3 against, **a** FMP013 antigen; **b** (NANP) × 6 repeat antigen. Individual animal and at 2 weeks post 3rd dose showing; **c** NPNA ELISA titers; **d** NPNA antibody avidity index; **e** inhibition of liver stage development (ILSDA) assay performed at 1:200 serum dilution (4 replicates per sample). Bars represent group mean  ±  SEM (n  =  6)

To compare the kinetics of induction of antibodies, group mean titers were compared between sub-populations over the course of vaccination. An ELISA against FMP013 coat antigen (Fig. 3a) and the NPNA repeat peptide (Fig. 3b) showed that all animals seroconverted following the first dose and subsequent vaccinations boosted titers. The titers against the FMP013 antigen were identical in the two sub-populations over time (Fig. 3a). However, there was a trend towards slower acquisition of antibodies in the ChR group against the NPNA repeat region (Fig. 3b). The InR group mean NPNA-specific titer at 2 weeks post first dose (19,036 vs. 7819), 4 weeks post first dose (20,468 vs. 7070) and 4 weeks post second dose (36,631 vs. 18,201), were higher than the ChR group (Fig. 3b). These differences in titer were however not statistically significant, and NPNA titer in both models reached near equivalence after the third dose (38,571 vs. 41,066). NPNA ELISA titers at 2 weeks post third dose were statistically equivalent between the two sub-populations (Fig. 3c), but the NPNA-specific avidity was higher in the ChR model (Fig. 3d). *P. falciparum* does not infect rhesus macaques and there are no transgenic parasites available to challenge rhesus monkeys. Instead, an in vitro inhibition of liver stage development assay (ILSDA) was used to compare antibody function. As was seen with avidity, the ILSDA activity, at 1:200 dilution, for FMP013 induced antibodies was higher in the ChR group than in InR (Fig. 3e). At 1:300 serum dilution the inhibition levels in both InR and ChR groups were < 5% (not plotted).

### T-cell activity

**Fig. 4:**
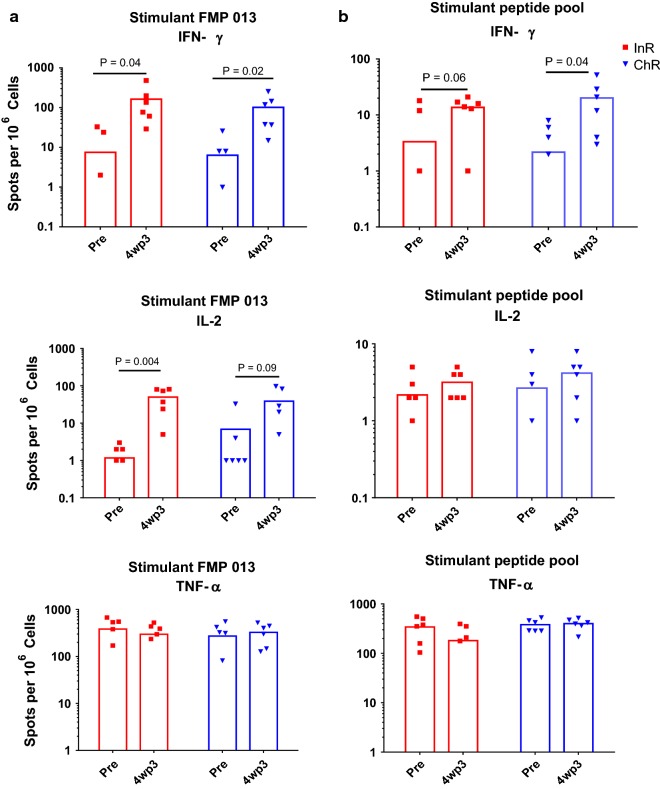
T-cell activity: at 4 weeks post 3rd vaccination T-cell responses induced by FMP013 vaccination were measured in InR and ChR by IFN-γ, IL-2 and TNF-α Flourospot assay. Rhesus PBMCs were stimulated with either **a** FMP013 antigen or **b** a pool of peptides spanning the full length CSP sequence. Bars represent mean  +  SEM spots formed per million cells (n  =  6)

PBMCs collected at 4 weeks post 3rd vaccine were stimulated with either the FMP013 protein or a CSP peptide pool that spans the repeats, N- and C-terminal regions (Fig. 4). InR and ChR both showed IFN-γ responses above pre-immune controls which were elicited by the FMP013 vaccine. Likewise, FMP013 vaccine induced IL-2 responses in both ChR and InR that could be recalled more readily by stimulating with the FMP013, but not the peptide pool, showing similar T-epitope usage by the two models. No TNF responses were observed. Overall, InR and ChR closely replicated the T-cell responses elicited by the FMP013 vaccine.

## Discussion

Mouse, rabbit and rhesus models have been used to down-select and predict immunogenicity and functional outcomes of malaria vaccine candidates. While mouse and rabbit models have not been good predictors of malaria vaccine success in humans [31–34], the rhesus model played a key role in the development and improvement of the most advanced malaria vaccine RTS,S/AS01. A preliminary study in InR led to switching the adjuvant for RTS,S to AS01B in humans [29, 35, 36]. Another rhesus study in InR showed that priming RTS,S with a dose of adenovirus vectored CSP could augment CD4+ cellular responses, which was subsequently reproduced in humans [37, 38]. There is evidence to suggest that HLA genotype may influence RTS,S-mediated protective efficacy [39]. While InR have been most widely employed for vaccine studies, functional characterization of the most common MHC class I allele among disparate ChR (Mamu-A1*02201), shows a significant overlap with the peptide binding repertoire of HLA-B7, the most frequent supertype in human populations [40]. ChR may at least as good if not better than InR for testing malaria vaccines. Indeed, ChR are increasingly utilized to study human pathogenic viruses like Marburg, Ebola, influenza and HIV [27, 41–45].

In order to study if responses to a malaria vaccine CSP in the InR model differed significantly from the ChR sub-population, a nearly full-length soluble protein (FMP013) adjuvanted with a liposomal adjuvant ALFQ was tested in the InR and ChR models. The two rhesus models had similar baseline haematology and biochemistry parameters; both models replicated a mild local and systemic adverse reaction to the vaccines, characterized by an elevation of CK (2000–5000 IU/L), transient neutrophilia and monocytosis. Elevation of CK has been indicated in striated muscle damage and co-elevation of neutrophils and monocytes are markers of systemic inflammation known to be associated with the administration of QS-21 containing adjuvants [29, 46]. These observations established that the FMP013/ALFQ vaccine was safe and that ChR and InR could closely replicate toxicological effects of vaccines.

FMP013 induced T-cell response and ELISA titer were similar in InR and ChR. However, vaccine-induced NPNA antibody titers increased more gradually in the ChR model and after the final vaccine dose, antibodies in the ChR group had higher NPNA-specific avidity and higher ILSDA activity than the InR model. Avidity of CSP antibodies has been associated with protection [30] and inhibition of sporozoite invasion by repeat-specific antibodies is believed to be critical for RTS,S mediated protection [47]. The observed differences in antibody responses between ChR and InR were not associated with the sex or weight of the animals. It cannot be completely rule out that the imported ChR used in this study had been naturally exposed to simian malaria and the observed difference in humoral response between InR and ChR was due to a pre-existing anti-malaria immunity in wild-caught ChR. It is notable that two independent HIV/SIV studies have also reported superior antibody titers and neutralizing activity were elicited in the ChR model [24, 45]. Due to the small sample size (n = 6), future studies with other CSP vaccines are needed to definitively establish if the two sub-populations of rhesus truly differ in their response to the repeat epitope of CSP.

The frontline CSP vaccine, RTS,S, is a particulate antigen, expressed in yeast and it contains only the major repeats and the C-terminal region of CSP. In contrast, FMP013 is nearly full-length, soluble protein, expressed in *E. coli*, and it contains key N-terminal and junctional epitopes that are not present in RTS,S [48, 49]. In mice and two sub-populations of rhesus, FMP013/ALFQ vaccine was found safe and it elicited antibody, T-cell responses that have been associated with RTS,S-mediated protection [10, 11, 35]. Positive outcome of a CHMI trial with FMP013/ALFQ vaccine can establish if epitope broadening can augment CSP vaccine efficacy [50]; if soluble proteins can still be considered a viable vaccine platform against malaria [51]; and provide the first evidence of safety and potency of a novel adjuvant ALFQ in humans [12].

## Conclusions

As new paradigms of vaccinology emerge, rhesus model can play a pivotal role in accelerating and de-risk preclinical selection of CSP-based vaccines, where comparison to a benchmark can be used to select improved vaccines. Based on comparisons to historical data on RTS,S/AS01, WRAIR Malaria Vaccine Branch has utilized an InR immunogenicity study to transition the FMP013/ALFQ vaccine to a Phase I human trial [10]. Keeping in mind some differences in antibody quality reported here, InR and ChR macaques could be used inter-changeably as a valid model for conducting down-selection studies.

## Supplementary information

**Additional file 1: Table S1.** Sex, weight and birthdate of all rhesus used within the study. **Table S2A, B.** Mean Blood Counts and Blood Chemistry data across each group on days 1, 3, 7 post vaccination.

## Data Availability

The datasets used and/or analyzed during the current study are available from the corresponding author at a reasonable request.

## Abbreviations

CSP: circumsporozoite protein
FMP013: falciparum malaria protein-013 comprising of CSP
ALFQ: army liposomal formulation containing QS21
InR: Indian origin rhesus macaques
ChR: Chinese origin rhesus macaques
WRAIR: Walter Reed Army Institute of Research
