# Supplementary material for: Comparison of immunogenicity and safety outcomes of a malaria vaccine FMP013/ALFQ in rhesus macaques (Macaca mulatta) of Indian and Chinese origin
Source: Malar J. 2019 Nov 27;18:377. doi: 10.1186/s12936-019-3014-5 (PMC6880475; doi:10.1186/s12936-019-3014-5)
Supplement: Supplementary file 1 — Additional file 1: Table S1. Sex, weight and birthdate of all rhesus used within the study. Table S2A, B. Mean Blood Counts and Blood Chemistry data across each group on days 1, 3, 7 post vaccination. [file 12936_2019_3014_MOESM1_ESM.pdf]

InR						ChR				
ID	Strain	Sex	Weight (kg)	Birthdate		ID	Strain	Sex	Weight (kg)	Birthdate
11C073	Indian	Male	10.38	29-Apr-11		142543	Chinese	Female	5.56	19-Feb-11
09U011	Indian	Female	8.1	23-Feb-09		142565	Chinese	Female	5.32	26-Mar-10
11D203	Indian	Male	6.54	19-Dec-11		66164	Chinese	Female	4.06	20-Feb-06
10D213	Indian	Female	6.04	17-Aug-10		RA0454	Chinese	Female	6.42	25-Apr-11
10C160	Indian	Male	10.76	11-Aug-10		142569	Chinese	Female	5.42	5-Feb-10
10U004	Indian	Female	5.44	12-Mar-10		RA0464	Chinese	Female	6.38	28-Apr-11

Additional file 1: Table S1

InR

CBC	Reference Range	Baseline	Vaccine 1 2/21/2018 Day 1	Vaccine 1 2/23/2018 Day 3	Vaccine 1 2/27/2018 Day 7	Vaccine 2 3/20/2018 Day 1	Vaccine 2 3/22/2018 Day 3	Vaccine 2 3/26/2018 Day 7	Vaccine 3 4/17/2018 Day 1	Vaccine 3 4/19/2018 Day 3	Vaccine 3 4/23/2018 Day 7
WBC (2.9 - 13.2)	4.00 - 13.00	6.31	15.10	7.45	7.76	13.24	7.51	6.70	12.22	6.65	7.81
RBC (4.1 - 7.8)	4.30 - 6.20	5.37	5.24	4.97	4.92	5.08	4.73	5.13	5.14	5.00	4.88
HGB	10.6 - 14.2	12.68	12.43	11.72	11.60	11.92	11.08	11.88	11.97	11.65	11.47
HCT	36.6 - 43.8	40.15	39.42	37.72	37.43	38.63	35.67	38.95	38.23	37.63	36.62
MCV	65.6 - 76.9	74.85	75.17	75.95	76.17	76.13	75.32	75.92	74.45	75.33	75.12
MCH	20.6 - 24.6	23.65	23.72	23.57	23.58	23.48	23.40	23.12	23.30	23.30	23.60
MCHC	30.6 - 32.8	31.60	31.52	31.03	30.95	30.83	31.03	30.43	31.25	30.95	31.40
PLT	97 - 436	348.83	366.67	336.17	432.83	357.17	351.00	500.83	341.17	327.17	430.00
MPV	9.2 - 12.4	11.18	11.25	11.32	10.92	11.25	11.37	10.80	11.33	11.50	11.18
#NEUT	1.25 - 9.20	2.64	12.18	4.03	4.04	10.58	4.64	2.72	10.20	3.40	3.59
#LYMPH	0.80 - 3.71	3.09	1.95	2.71	3.03	1.89	2.41	3.48	1.43	2.63	4.02
#MONO	0.13 - 0.63	0.45	0.90	0.61	0.56	0.65	0.64	0.47	0.54	0.56	0.54
#EOS	0.00 - 0.18	0.13	0.07	0.08	0.10	0.11	0.08	0.03	0.04	0.06	0.02
#BASO	0.00 - 0.01	0.01	0.02	0.02	0.02	0.01	0.01	0.01	0.01	0.01	0.01
Chemistry	-										
BUN	11 - 27	14.67	14.00	14.33	14.33	12.67	12.17	14.17	13.17	12.17	14.83
Creatinine	.3 - 1.4	0.88	0.90	0.90	0.87	0.92	0.90	0.85	0.87	0.88	0.80
Total Protein	5.8 - 7.4	6.78	6.78	6.68	6.70	6.65	6.47	6.93	6.57	6.62	6.82
AST	29 - 64	61.33	53.33	49.67	40.33	64.17	37.17	28.83	63.33	40.83	28.33
ALT	19 - 91	61.33	41.83	54.33	54.83	51.67	50.33	41.17	55.50	55.00	41.50
LDH	639 - 2903	1213.17	1229.33	978.67	1077.83	1080.17	675.67	633.00	1045.00	811.00	599.67
CK	69 - 972	421.00	1032.33	545.00	696.17	1721.83	555.50	398.33	1574.17	422.00	260.67
ALKP	65 - 400	122.83	131.00	129.17	115.33	132.67	133.00	124.17	118.50	114.83	106.50
Total Bili	.1 - .4	0.10	0.25	0.17	0.23	0.20	0.18	0.18	0.15	0.15	0.13

Additional file 1: Table S2A

ChR

			Vaccine 1			Vaccine 2			Vaccine 3		
			2/21/2018	2/23/2018	2/27/2018	3/20/2018	3/22/2018	3/26/2018	4/17/2018	4/19/2018	4/23/2018
CBC	Reference Range	Baseline	Day 1	Day 3	Day 7	Day 1	Day 3	Day 7	Day 1	Day 3	Day 7
WBC (2.9 - 13.2)	4.00 - 13.00	9.54	16.41	8.78	7.92	13.29	6.72	5.75	11.46	6.05	8.52
RBC (4.1 - 7.8)	4.30 - 6.20	4.89	4.89	4.58	4.60	4.71	4.43	4.69	4.85	4.56	4.81
HGB	10.6 - 14.2	11.80	11.82	11.07	11.05	11.25	10.52	11.05	11.50	10.80	11.30
HCT	36.6 - 43.8	36.92	37.35	35.48	35.53	36.48	34.57	35.85	36.45	34.78	36.37
MCV	65.6 - 76.9	75.62	76.55	77.60	77.35	77.75	78.08	76.58	75.38	76.45	75.88
MCH	20.6 - 24.6	24.17	24.20	24.17	24.02	23.95	23.75	23.62	23.73	23.68	23.55
MCHC	30.6 - 32.8	31.95	31.65	31.20	31.12	30.80	30.45	30.83	31.53	31.05	31.05
PLT	97 - 436	357.17	357.50	329.67	415.00	357.17	369.00	532.83	386.83	375.00	451.67
MPV	9.2 - 12.4	10.72	10.87	11.10	10.75	10.87	10.97	10.40	10.65	10.97	10.80
#NEUT	1.25 - 9.20	6.65	13.40	4.90	4.56	11.10	3.47	2.36	9.57	3.04	4.98
#LYMPH	0.80 - 3.71	2.44	1.90	3.01	2.83	1.44	2.58	2.82	1.29	2.40	2.93
#MONO	0.13 - 0.63	0.36	1.01	0.72	0.38	0.65	0.48	0.37	0.52	0.45	0.45
#EOS	0.00 - 0.18	0.08	0.08	0.14	0.12	0.10	0.19	0.20	0.07	0.15	0.16
#BASO	0.00 - 0.01	0.02	0.02	0.02	0.03	0.01	0.01	0.01	0.01	0.01	0.01
Chemistry	-										
BUN	11 - 27	13.83	13.33	14.33	13.50	11.33	12.33	13.33	10.33	13.50	14.50
Creatinine	.3 - 1.4	13.83	0.75	0.72	0.75	0.75	0.68	0.73	0.67	0.62	0.62
Total Protein	5.8 - 7.4	6.95	7.42	7.07	7.19	7.23	6.70	7.43	6.87	6.77	7.22
AST	29 - 64	34.17	78.67	58.50	47.67	48.33	30.83	30.17	47.50	41.83	30.50
ALT	19 - 91	49.00	57.83	86.50	85.83	36.50	40.50	44.83	45.67	50.50	48.17
LDH	639 - 2903	525.67	838.17	646.00	559.83	750.83	502.67	513.67	702.50	751.17	559.67
CK	69 - 972	235.83	1099.67	273.33	308.67	766.00	304.83	326.17	701.67	391.33	272.17
ALKP	65 - 400	123.17	144.83	153.00	141.33	158.00	151.17	136.33	152.83	136.17	144.50
Total Bili	.1 - .4	0.22	0.28	0.17	0.12	0.22	0.15	0.15	0.18	0.18	0.18

Additional file 1: Table S2B
